# FROM STRESSED CAREGIVERS TO HEALTHY CAREGIVING: THE IMPLICATIONS OF BALANCED PERSPECTIVES AND UPDATED FINDINGS

**DOI:** 10.1093/geroni/igac059.585

**Published:** 2022-12-20

**Authors:** David Roth

**Affiliations:** Johns Hopkins University, Baltimore, Maryland, United States

## Abstract

Research on family caregiving continues to evolve and stress process models are now frequently balanced by perspectives of benefits emanating from prosocial behaviors including caregiving and volunteering. Initial findings that caregivers have elevated inflammation levels and shorter life expectancies than non-caregivers have been contradicted by numerous more recent findings from larger, population-based, epidemiological studies. In many ways, the caregiving literature shows a bias pattern that is sometimes found in other areas, where initial studies with relatively small samples and alarming results are widely cited, whereas subsequent studies with larger samples and contradicting results are given much less attention. A minority of caregivers are highly stressed, but most caregivers are resilient and face other challenges besides stress-related health problems. Caregivers are the backbone of long-term care, and interventions and policy initiatives to support caregivers are vital, but should be based on replicable findings of verifiable challenges to vulnerable caregiving subgroups.

